# Supplementary material for: COVID-19 Vaccine Education (CoVE) for Health and Care Workers to Facilitate Global Promotion of the COVID-19 Vaccines
Source: Int J Environ Res Public Health. 2022 Jan 7;19(2):653. doi: 10.3390/ijerph19020653 (PMC8775929; doi:10.3390/ijerph19020653)
Supplement: Supplementary file 1 [file ijerph-19-00653-s001.zip › Supp Files/File S4_Interview topic guide.pdf]

Supplementary File S4. Interview Topic Guide

Supplementary File S4: Interview topic guide

Level 1: Reaction

(satisfaction) What is your overall view of this resource?

(engagement) When using the resource, what is your view of the interactive elements? (by this we mean: menu, narration adjustments (can turn on or off and change the speed), video clips, 'i' information boxes, click boxes, quiz, extra resources).

(relevance) How relevant is this resource to you? Or to others? Will you have the opportunity to use this information or apply it in your studies or job?

Level 2: Learning

(Knowledge) Did you learn anything new from using this resource?

(Skill) Did this resource equip you with useful knowledge about the COVID-19 vaccine?

(Attitude) Do you think the COVID-19 vaccine is important for individual and societal health? Have you changed any of your views after using this resource?

(Confidence) Do you feel more confident to talk to other people (patients or clients) about the importance of the COVID-19 vaccine after using this resource?

(Commitment) Are you likely to use any of this information in the future? If so, how? Do you intend to share the resource? Tell us more.

Level 3: Behaviour

(Behaviour) Have you applied any of the knowledge from this resource in your life, studies or job? If so, what and how? Have you done anything differently?

(Required drivers) How do you think this resource should be best used? Who should use it and how is it best to provide them with access to it?

Level 4: Results

(Leading indicators) Have there been any other benefits of using or sharing this resource?
If you have applied this knowledge (e.g. by talking to others about the COVID-19 vaccine), do you think it has made any difference? To what degree? (prompt: have you been able to answer people's questions better, or signpost people better, or changed anyone's mind about the importance of the vaccine? Has anyone indicated that they have taken a vaccine based on your communication with them after using this resource?
