# Supplementary material for: COVID-19 Vaccine Education (CoVE) for Health and Care Workers to Facilitate Global Promotion of the COVID-19 Vaccines
Source: Int J Environ Res Public Health. 2022 Jan 7;19(2):653. doi: 10.3390/ijerph19020653 (PMC8775929; doi:10.3390/ijerph19020653)
Supplement: Supplementary file 1 [file ijerph-19-00653-s001.zip › Supp Files/File S1_Stage 1 specification review form.pdf]

Reusable Learning Object Peer Review (1) - Specification

Reusable Learning Object to be reviewed:

Title | | RLO ID | |

RLO Author:

Name | | Phone number | |

E-mail | |

Reviewer:

Name | |

Date requested | | Date review required | |

Reviewer's instructions

Please read through the specification sheet and any additional materials supplied. Wherever possible, please contact the Author to discuss your comments and suggested revisions, then complete this form's 11 questions (including the tick boxes about revision). The text boxes will expand as you write but if you wish to include additional sheets, please label them with the RLO ID number.

Once completed, please return this form by the date shown to the person named below

Author's instructions,

Please use the boxes labelled Author's revisions to respond to the reviewer's comments. You may wish to submit a revised Specification and then cross reference the changes onto this form.

Once completed, please return this form to the person named below

Name

Address

E-mail

1) Is the learning objective clear and do all sections of the RLO support it?

Is Revision Required? **Yes** ☐ **No** ☐

Author's revisions

2) Is the content factually correct?

Is Revision Required? **Yes** ☐ **No** ☐

Author's revisions

3) Is the text well written in short, clear, sentences?

Is Revision Required? **Yes** ☐ **No** ☐

Author's revisions

4) Does the glossary cover all the terms required for a general audience?

Is Revision Required? **Yes** ☐ **No** ☐

Author's revisions

5) Is the structure and sequence of information helpful?

Is Revision Required? Yes ☐ No ☐

Author's revisions

6) Are the suggestions/examples for images/animations/video appropriate?

Is Revision Required? Yes ☐ No ☐

Author's revisions

7) Is sufficient interaction proposed to support active learning?

Is Revision Required? Yes ☐ No ☐

Author's revisions

8) Will the assessments measure attainment of the learning objective?

Is Revision Required? Yes ☐ No ☐

Author's revisions

9) Are the keywords appropriate? Are others needed?

Is Revision Required? **Yes** ☐ **No** ☐

Author's revisions

10) Are the suggested links OK? Are there others that you could suggest?

Is Revision Required? **Yes** ☐ **No** ☐

Author's revisions

11) Have you discussed your review with the authors? **Yes** ☐ **No** ☐

Nature of communication (eg face-to-face, e-mail etc)

Additional comments or continuations of above sections.

Please continue on additional sheets as required
