# Supplementary material for: COVID-19 Vaccine Education (CoVE) for Health and Care Workers to Facilitate Global Promotion of the COVID-19 Vaccines
Source: Int J Environ Res Public Health. 2022 Jan 7;19(2):653. doi: 10.3390/ijerph19020653 (PMC8775929; doi:10.3390/ijerph19020653)
Supplement: Supplementary file 1 [file ijerph-19-00653-s001.zip › Supp Files/File S3_Survey items.pdf]

Supplementary File S3. Survey items

Pre-Survey: Embedded questions

Before we start:
How do you rate your current knowledge about the COVID-19 vaccine?
Not at all knowledgeable Extremely knowledgeable
1 2 3 4 5 6 7 8 9 10
How confident are you in communicating the importance of the COVID-19 vaccine to others?
Not at all confident Extremely confident
1 2 3 4 5 6 7 8 9 10

Post-Survey: Embedded Evaluation Form

1. How did you rate your knowledge about the COVID-19 vaccine prior to completing this resource? Rate between 1 (Not at all knowledgeable) and 10 (Extremely knowledgeable).

2. How did you rate your confidence in communicating the importance of the COVID-19 vaccine to others prior to completing this resource?
Rate between 1 (Not at all confident) and 10 (Extremely confident).

3.How do you rate your current knowledge about the COVID-19 vaccine?
Please don't select more than 1 answer(s) per row.

1 2 3 4 5 6 7 8 9 10
Not at all knowledgeable Extremely knowledgeable

4.Have you learned something new from this resource? Required
Yes / No

5.How confident are you in communicating the importance of the COVID-19 vaccine to others?
1 2 3 4 5 6 7 8 9 10
Not at all confident Extremely confident

6. How would you rate this learning object?
Excellent/ Good/ Not very good/ Poor

7. How easy was it to use the learning object?
Very easy/ Easy/ Not very easy/ Difficult

8.How helpful has the resource been for learning this subject?
Very helpful/ Helpful/ Unhelpful/ Very Unhelpful

9.What was your main reason for using this resource?

10. Are you: (tick all that apply)
Healthcare professional
University or college student (further and higher education)
School student (up to age 16)
Tutor / Teacher / Lecturer

General public
Other

11. How did you find out about this resource? (tick all that apply)
Through my employer
Through my educational institution
Via a professional network
Recommended by a colleague/peer
Through HELM-Open
Through Open Educational Resource (OER) catalogues
General internet search
Other

12.Did you experience any of these problems in using this resource?
No problems experienced
Technical
Level of difficulty/study
Language
Contextual/cultural differences
Other

13.Would you recommend it to others?
Yes/No

14.What did you most like about this resource?

15.What did you least like about this resource? R

16. In order to help us track use of this resource, please let us know what country and organisation / institution you are from.

Participant could provide contact details to provide more feedback (optional).
