# Supplementary material for: COVID-19 Vaccine Education (CoVE) for Health and Care Workers to Facilitate Global Promotion of the COVID-19 Vaccines
Source: Int J Environ Res Public Health. 2022 Jan 7;19(2):653. doi: 10.3390/ijerph19020653 (PMC8775929; doi:10.3390/ijerph19020653)
Supplement: Supplementary file 1 [file ijerph-19-00653-s001.zip › Supp Files/File S2_Stage 2 media review forms.pdf]

# Reusable Learning Object Peer Review (2) - Media

**Reusable Learning Object to be Reviewed:**

Title | RLO ID

URL

**RLO Author:**

Name | Phone number

E-mail

**RLO Developer:**

Name | Phone number

Email

**Reviewer:**

Name

Phone Number | E-mail

Date requested | Date review required

## Reviewer's instructions

Please work through the RLO at the URL address above, then complete the 9 questions (including the tick boxes about revision). The boxes will expand as you write.

**Once completed, please return it by the date shown to the person named below**:

## Author's instructions

Please use the boxes labeled ''Author's revisions'' to note any amendments that you wish to make in response to the Reviewer's comments.
You should discuss these with the RLO Developer and/or Mentor before proceeding.

**Please return the form to the person named below and arrange to discuss the suggested revisions with your developer.**

Name

Address

E-mail

**1) Is the general look & "feel" of the RLO coherent and conducive for learning?**

**Is Revision Required?** **Yes** ☐ **No** ☐

Author's revisions

**2) How long would it take students to complete this RLO, in your estimation?**

**Is Revision Required?** **Yes** ☐ **No** ☐

Author's revisions

**3) Is the narration clear, audible and engaging?**

**Is Revision Required?** **Yes** ☐ **No** ☐

Author's revisions

**4) Do the animations/images/video support the learning of this subject?**

**Is Revision Required?** **Yes** ☐ **No** ☐

Author's revisions

**5) Are the interactions appropriate and engaging?**

**Is Revision Required?** **Yes** ☐ **No** ☐

Author's revisions

**6) Is the assessment effective & engaging as a test of the learning objective?**

**Is Revision Required?** **Yes** ☐ **No** ☐

Author's revisions

**7) Is the RLO easy to navigate?**

**Is Revision Required?** **Yes** ☐ **No** ☐

Author's revisions

**8) Would this RLO be useful for a range of courses?**

**9) Have you discussed your review with the authors?** **Yes** ☐ **No** ☐

**Nature of communication (eg face-to-face, e-mail etc)**
